# Gibberellin-like effects of KAR_1_ on dormancy release of *Avena fatua* caryopses include participation of non-enzymatic antioxidants and cell cycle activation in embryos

**DOI:** 10.1007/s00425-015-2422-1

**Published:** 2015-11-02

**Authors:** Danuta Cembrowska-Lech, Jan Kępczyński

**Affiliations:** Department of Plant Physiology and Genetic Engineering, Faculty of Biology, University of Szczecin, Wąska 13, 71-415 Szczecin, Poland

**Keywords:** ABA catabolism, Ascorbate peroxidase, DNA replication, Glutathione reductase, Karrikinolide, Phaseic acid, β-Tubulin

## Abstract

*****Main conclusion***:**

**The induction of dormancy release and germination of*Avena fatua*caryopses by KAR**_**1**_**involves ABA degradation to phaseic acid. Both, KAR**_**1**_**and GA**_**3**_**, control the AsA–GSH cycle, DNA replication and accumulation of β-tubulin in embryos before caryopses germination.**

**Abstract:**

*Avena fatua* caryopses cannot germinate in darkness at 20 °C because of dormancy, but karrikinolide-1 (KAR_1_), a compound in plant-derived smoke, and gibberellic acid (GA_3_) induced an almost complete germination. The radicle protrusion through the coleorhiza was preceded by increased water uptake, rupture of coat, increased embryo size and coleorhiza length as well as coleorhiza protrusion through covering structures. The stimulatory effect of KAR_1_ was correlated with the reduced content of abscisic acid (ABA) and an increase in phaseic acid (PA) in embryos from caryopses before coleorhiza protrusion. Two non-enzymatic antioxidants, ascorbate (AsA) and reduced glutathione (GSH), did not affect the germination of dormant caryopses, but in the presence of KAR_1_ or GA_3_ they only slightly delayed the germination. The stimulatory effect of KAR_1_ or GA_3_ on the final germination percentage was markedly antagonized by lycorine, an AsA biosynthesis inhibitor. KAR_1_ and GA_3_ applied during caryopses imbibition resulted in increases of AsA, dehydroascorbate (DHA) and GSH, but reduced the embryos’ oxidized glutathione (GSSG) content. Furthermore, both KAR_1_ and GA_3_ induced an additional ascorbate peroxidase (APX) isoenzyme and increased the glutathione reductase (GR) activity. Both compounds stimulated β-tubulin accumulation in radicle+coleorhiza (RC) and plumule+coleoptile (PC), and enhanced the transition from G_1_ to S and also from S to G_2_ phases. The comparison of the effects produced by KAR_1_ and GA_3 _ shows a similar action; thus the KAR_1_ effect may not be specific. The study provides new data regarding the mechanism with which KAR_1_, a representative of a novel class of plant growth regulators, regulates dormancy and germination of caryopses.

## Introduction

Seed dormancy is induced during seed maturation and is defined as the inability of intact viable seeds to germinate under conditions favorable for the germination process (Bewley et al. 2013). Abscisic acid (ABA)  is considered to play a central role in the induction and maintenance of seed dormancy. The degree of seed dormancy is correlated with the ABA content in imbibed seeds. Many studies have shown that transition from dormancy to germination requires catabolic removal of ABA by hydroxylation and/or conjugation (Rodríguez-Gacio et al. 2009). Mutual antagonism between ABA and gibberellins (GAs) associated with biosynthesis and signaling is known. It is well documented that the balance between synthesis and catabolism of ABA and GAs and the sensitivity to these hormones are mainly responsible for dormancy status in seeds. ABA is responsible for induction and maintenance of dormancy and GAs promote the progression from release through germination (Finkelstein et al. 2008; Rodríguez-Gacio et al. 2009). Participation of other hormones, such as ethylene, cytokinins and brassinosteroids in releasing seed dormancy and in germination, has been also discussed (Kępczyński and Kępczyńska 1997; Finch-Savage and Leubner-Metzger 2006; Finkelstein et al. 2008). Likewise, reactive oxygen species (ROS), superoxide (O_2_^•−^), hydrogen peroxide (H_2_O_2_), hydroxyl radical (HO∙) and singlet oxygen (^1^O_2_) play important roles in the seed life (Bailly et al. 2008). These compounds have detrimental effect on, and also play a key signaling function in, germination and dormancy release. Germination can be only completed when the ROS concentration is suitable and its content is within the ‘oxidative window for germination’ (Bailly et al. 2008). From among ROS, the role of H_2_O_2_ is most important, since H_2_O_2_ can remove dormancy in seeds of several dicotyledonous and monocotyledonous species (Diaz-Vivancos et al. 2013). Because of this dual function, the ROS content in cells must be tightly regulated by the balance between production and scavenging by non-enzymatic and enzymatic antioxidants. Non-enzymatic antioxidants are represented by α-tocopherol, the reduced glutathione (GSH) and ascorbate (AsA); the enzymatic antioxidants involve superoxide dismutase (SOD) converting O_2_^•−^ to H_2_O_2_, catalase (CAT) converting H_2_O_2_ to water and oxygen, as well as the enzymes cooperating with the ascorbate–glutathione cycle: ascorbate peroxidase (APX), monodehydroascorbate reductase (MR), dehydroascorbate reductase (DHAR) and glutathione reductase (GR) (Noctor and Foyer 1998).

The ascorbate–glutathione pathway is involved in indirect or direct scavenging of ROS (Foyer and Noctor 2011). Numerous authors have demonstrated the ascorbate–glutathione cycle to be mainly responsible for scavenging of H_2_O_2_ (Li et al. 2010). APX detoxifies H_2_O_2_ using AsA as electron donor. AsA is regenerated by MR and DHAR, with participation of GSH and NADPH. GSH is oxidized to oxidized glutathione (GSSG) and can be reduced by glutathione reductase (GR) to GSH (Li et al. 2010).

Information on involvement of the ascorbate–glutathione cycle in regulation of seed dormancy and germination is scant. A rapid increase in contents of AsA and APX activity was observed during initial hours of imbibition of non-dormant seeds (De Tullio and Arrigoni 2003). AsA has also been demonstrated to be able to suppress germination of wheat seeds (Ishibashi and Iwaya-Inoue 2006). It was suggested as early as in 1994 that glutathione is included in dormancy alleviation of barley seeds (Fontaine et al. 1994). Recently, GSH has been reported to be gradually converted to GSSG during after-ripening of barley grains (Bahin et al. 2011). After-ripening did not affect GR activity in barley caryopses (Bahin et al. 2011) and slightly decreased the enzyme’s activity in *Arabidopsis* seeds during their imbibition (Leymarie et al. 2012). Experiments with pea seeds (Wojtyla et al. 2006) showed GR to be activated during germination. GR activity was found to increase prior to radicle protrusion in sunflower seeds (Oracz et al. 2009).

Scant information is available on the cell cycle in relation to dormancy release, germination and their regulation by AsA and GSH. Cells of embryos from imbibed dormant tomato seeds have been shown to remain in the G_1_ phase until dormancy was released (de Castro et al. 2001). The redox state is considered to play a crucial regulating function in the cell cycle (Foyer and Noctor 2011). On the basis of numerous studies, it has been postulated that glutathione is a key regulator of cell proliferation. Plant cells in phase G_1_ of the cell cycle have very low GSH level; for the cells to progress from G_1_ to S phase an increase in total GSH is necessary (Kerk and Feldman 1995). Ascorbate has been shown to stimulate the cell cycle of cells from a root tip of the quiescent center of *Allium cepa* by shortening G_1_ and stimulating the entry into the S phase (Liso et al. 1984). Data are available showing that germination completion (radicle protrusion) does not require mitotic activity (Baiza et al. 1989); there is also evidence that cell division proceeds prior to radicle protrusion (Masubelele et al. 2005).

Germination of dormant and non-dormant seeds can be stimulated by hormones such as GAs, ethylene, cytokinins and brassinosteroids as well as by plant-derived smoke and butenolide. Butenolide (3-methyl-2*H*-furo[2,3-*c*]pyran-2-one), at present termed karrikinolide-1 or karrikin-1 (KAR_1_) (Dixon et al. 2009), was isolated from plant-derived smoke (Van Staden et al. 2004) and burned cellulose (Flematti et al. 2004). Most papers on seeds describe effects of KAR_1_ on germination only. Karrikins, KAR_1_ and KAR_2_-KAR_6_, also found in smoke (Flematti et al. 2009), were considered as a novel family of regulators; it was even suggested that these compounds may be endogenous plant hormones which await identification (Nelson et al. 2009). *Avena fatua* caryopses have already been used in our studies as a model system to study the role of regulators, particularly that of KAR_1_, in the control of dormancy release and germination. *Avena fatua*, an annual grass, is worldwide distributed, mainly in cereal crops. Dormant caryopses can germinate after dry storage (Foley 1994; Kępczyński et al. 2013) or after gibberellic acid (GA_3_) treatment (Adkins et al. 1986; Kępczyński et al. 2006). In a previous study, we showed that smoke water, KAR_1_ and GA_3_ stimulate germination of dormant caryopses covered by lemma and palea and caryopses of *A. fatua* (Kępczyński et al. 2013). Likewise, H_2_O_2_, generating O_2_^•−^, and inhibiting catalase activity, induced germination of dormant caryopses (Cembrowska-Lech et al. 2015). The stimulatory effect of KAR_1_, GA_3_ and H_2_O_2_ on germination of *A. fatua* dormant caryopses was associated with a reduction of ABA content in embryos. Germination induction of *A. fatua* dormant caryopses by both KAR_1_ and GA_3_ was related to an increasing content of H_2_O_2_ and O_2_^•−^ and activities of enzymatic antioxidants, SOD and CAT in embryos. Thus, the ROS–antioxidant balance in embryos was probably required for the germination of dormant caryopses.

Until now, the role of non-enzymatic antioxidants, AsA and GSH, mainly has been studied in relation to germination of non-dormant, aged, stressed or primed seeds. So far, only few reports on glutathione in connection with dormant barley (Fontaine et al. 1995; Bahin et al. 2011), *Helianthus annuus* (Oracz et al. 2009) and *Lolium rigidum* (Goggin et al. 2011) seeds have been available. There are no data on cooperation of AsA or GSH with hormones stimulating germination of dormant seeds. Few papers have been published on hormone (mainly ABA) regulation of β-tubulin and/or cell cycle activity in relation to dormant tomato seeds (de Castro et al. 2001) and dormant barley grains (Gendreau et al. 2012).

Little information can also be found on the interaction of KAR_1_ with hormones in the regulation of germination of dormant seeds; only seeds of *Arabidopsis thaliana* (Nelson et al. 2009) and *A. fatua* (Kępczyński and Van Staden 2012; Kępczyński et al. 2013) have been studied. It is unknown whether induction of germination in dormant caryopses by KAR_1_ is associated with control of ABA metabolism and non-enzymatic antioxidants such as AsA and GSH. The participation of GAs in regulation of dormancy and germination of cereal and weed caryopses in relation to non-enzymatic antioxidants such as AsA and GSH has also not been studied. Likewise, participation of KAR_1_ and GAs in regulation of β-tubulin accumulation in relation to the cell cycle activity in dormant caryopses is unknown.

Taking into account the available information, the present study was conducted to examine: (I) whether induction of germination in dormant *A. fatua* caryopses by KAR_1_ is related to control in embryo exerted by ABA catabolism and (II) whether releasing dormancy and germination by KAR_1_ and also by GA_3_ involves regulation of (1) contents of non-enzymatic antioxidants, AsA, dehydoxyascorbate (DHA), GSH and GSSG, (2) cell cycle activity in radicle+coleorhiza (RC) and plumule+coleoptile (PC) and (3) β-tubulin accumulation. The effect of KAR_1_ or GA_3_ in combination with ASA, GSH or lycorine, an inhibitor of AsA biosynthesis, on germination of dormant caryopses was also explored.

## Materials and methods

### Plant materials

*Avena fatua* L. (wild oat) spikelets were collected in July 21, 2010, during the time of their natural dispersal, in the vicinity of Szczecin (Poland). The spikelets contained two to three florets covered with glumes. Each floret was a single caryopsis (fruit) covered by the lemma and palea (Simpson 2007). After collection, the florets were left to dry at room temperature for 7 days to a constant moisture content (ca. 11 %) and then stored at −20 °C until needed. The experiments involved the caryopses or embryos.

### Karrikinolide (KAR_1_) synthesis

KAR_1_ (3-methyl-2*H*-furo[2,3-*c*]pyran-2-one) was synthesized as described by Nagase et al. (2008). Briefly, the compound was synthesized using direct and region-selective Ti-cross aldol addition involving dihydro-2*H*-pyran-3-(4H)-one and methyl pyruvate as the keep step, followed by furanone formation. The crude product was purified with silica gel column chromatography to obtain a crystal form. The crystal structure was confirmed by ^1^H nuclear magnetic resonance (NMR) and high-resolution mechanical spectroscopy (HRMS). NMR spectra were recorded on a Bruker AC 200 spectrometer operating at 200.13 MHz (with CD13 as a solvent). MS spectra including HRMS were recorded on a Finnigan MAT 95 spectrometer.

### Germination assays

**Fig. 1:**
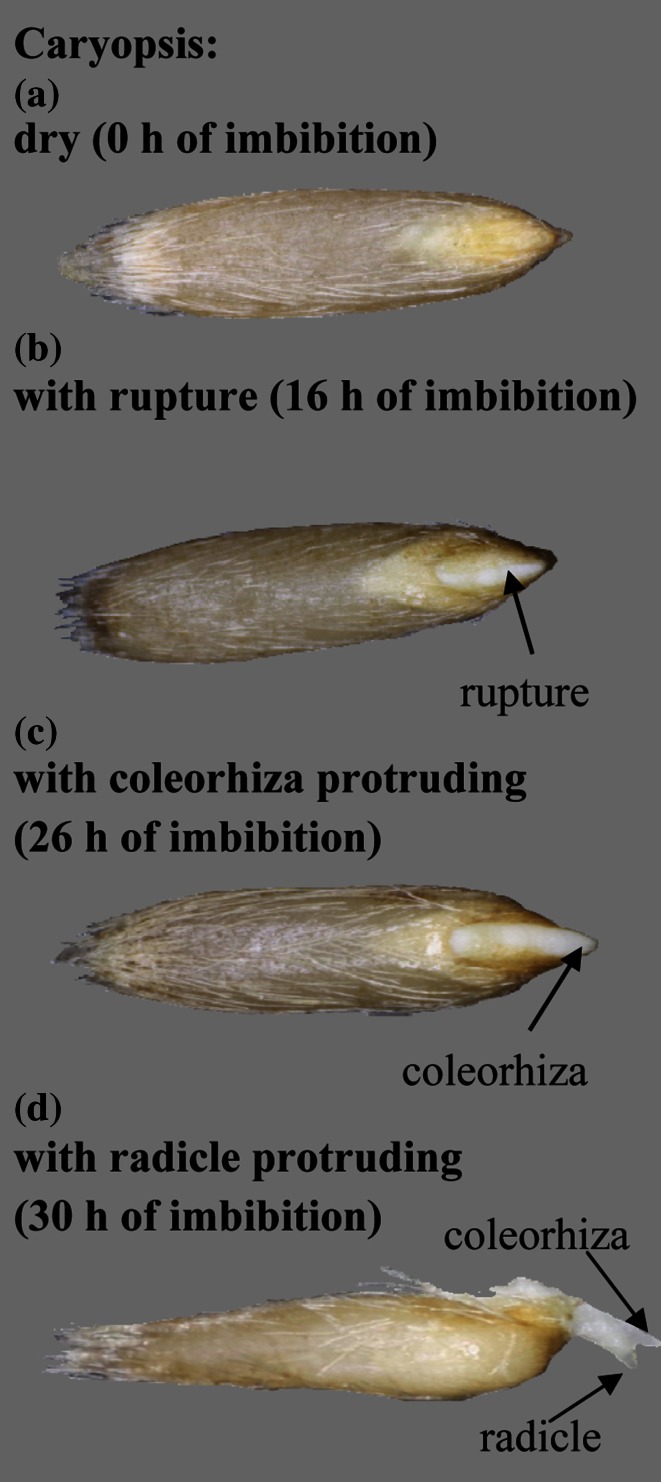
Germination of *A. fatua* caryopses. Caryopses were incubated in the presence of KAR_1_ at 20 °C for 30 h. Caryopsis: ungerminated (**a**), with coat rupture (**b**), with coleorhiza penetrating the coat (**c**), with radicle penetrating the coleorhizae (**d**). These three stages (Fig. 2) or last stage (Table 3; Fig. 5) was determined

Caryopses, 25 in 3 replicates, were incubated at 20 °C in the dark, in 6-cm Petri dishes on a single layer of filter paper (Whatman No. 1) moistened with 1.5 ml deionized water, KAR_1_ (3 × 10^−9^ M) or GA_3_ (10^−5^ M), either alone or in combination with AsA (10^−3^ M), GSH (10^−3^ M) or lycorine, an AsA biosynthesis inhibitor (5 × 10^−6^, 10^−5^, 2.5 × 10^−5^, 5 × 10^−5^, 10^−4^ or 2 × 10^−4^ M). Caryopses with ruptured coat, caryopses with the coleorhiza protruding through the coat and with a radicle protruding over the coleorhiza (Fig. 1) were examined at 4 h intervals up to the total duration of 28 h. After 2 and 5 days of incubation, only radicle protrusion was recorded (cf. Table 3; Fig. 5).

### Moisture content and dry weight determination

Dry weight (DW) of caryopses, 25 in 3 replicates, was determined gravimetrically on fully dried (105 °C for 24 h) specimens. Throughout this paper, the water content (WC) is expressed on the fresh weight (FW) basis to represent the percentage of water in the total mass, and calculated as WC = [(FW - DW)/FW] × 100.

### Embryo size and coleorhiza length measurements

The embryos, 25 in 3 replicates, were isolated from the caryopses after imbibition, taking various periods of time, in H_2_O, KAR_1_ (3 × 10^−9^ M) or GA_3_ (10^−5^ M) solution. They were then transferred onto a dark board and photographed (Canon EOS 500). All the images were saved as TIFF files, with 3072 × 2304 pixel resolution and 24 bits RGB color depth (pixel transformation factor = 1, no scaling of result images). The image analysis was conducted using the Fiji ImageJ software v. 1.48a (Schindelin et al. 2012). The Threshold plugin used (from version 1.15) allowed to apply histogram-based cutting algorithms. Image binarization was performed with the default algorithm (‘Default’ menu option), a variation of the IsoData method. The threshold algorithm configuration was based on the available histograms: Histogram red: true; Histogram green: true; Histogram blue: true; Threshold method: Default; Threshold color: red; Color Space: RGB; Dark background: true. The histogram parameters were defined in a manner excluding possible artifacts from the measurements. A mask was thus created to cover the embryo surface, and simultaneously serving as an indicator of the area measured (Region of Interest, ROI). With the help of the Area Manager available in the Fiji (Region of Interest Manager), the embryo area to be measured, defined by the mask, was identified. The image garnering and processing settings listed above allowed to calculate the true area size of 1 square pixel on the image of the embryo being examined. The same embryo images were used for the coleorhiza length measurements using the Fiji ImageJ software v. 1.48a. Coleorhiza elongation was monitored by measuring the distance the coleorhiza tip had grown past the end of the scutellum.

### Determination of abscisic acid (ABA) and its metabolites

To quantify the endogenous level of ABA and its metabolites, phaseic acid (PA), dihydrophaseic acid (DPA), neophaseic acid (neoPA) and 7′hydroxyABA (7′OHABA) in embryos of *A. fatua* L. caryopses, 25 caryopses in 3 replicates were incubated at 20 °C in the dark, in 6-cm Petri dishes on a single layer of filter paper (Whatman No. 1) moistened with 1.5 ml deionized water or with the solution of 3 × 10^−9^ M KAR_1_. After 20 h, the embryos were dissected, frozen immediately in liquid nitrogen and stored at −80 °C prior to ABA assay. The ABA content and its metabolites were determined as described by Turečková et al. (2009). Twenty-five embryos were ground to a fine powder in liquid nitrogen using a Retsch MM200 laboratory mill ball, homogenized and extracted for 1 h in 1 ml ice-cold methanol/water/acetic acid (10/89/1, by vol.). A mixture of internal standards containing 50 pmol of each (+)-3′,5′,5′,7′,7′,7′-^2^H_6_-ABA, (−)-7′,7′,7′-^2^H_3_-phaseic acid, (−)-7′,7′,7′-^2^H_3_-dihydrophaseic acid, (−)-8′,8′,8′-^2^H_3_-neophaseic acid, (+)-4,5,8′,8′,8′-^2^H_5_-ABAGE and (−)-5,8′,8′,8′-^2^H_4_-7′-OH-ABA was added to the samples. The solutions were centrifuged (21,000*g*, 10 min, 4 °C) and the pellets were re-extracted, following an identical procedure, for further 30 min. The combined extracts were purified by solid-phase extraction on Oasis^®^ HLB cartridges (60 mg, 3 ml, Waters, Milford, MA, USA), evaporated to dryness in a Speed-Vac (UniEquip), and finally analyzed by UPLC-ESI(−/+)-MS/MS.

### Determination of nuclear DNA contents and detection of β-tubulin

The nuclear DNA contents in radicle with coleorhiza (RC) or in plumule with coleoptile (PC) were determined using flow cytometry. For cell-cycle activity determination, caryopses, 25 in 5 replicates, were incubated at 20 °C for 0, 24, 26 and 28 h in the dark in distilled water, KAR_1_ (3 × 10^−9^ M) or GA_3_ (10^−5^ M) solution. RCs (25) or PCs (25) were isolated from the imbibed caryopses and, using a razor blade, were chopped and placed in 2 ml of a nucleus isolation buffer (45 mM MgCl_2_, 30 mM sodium citrate, 20 mM MOPS, 0.1 % Triton X-100 and 2 µg/ml DAPI) (Galbraith et al. 1983) for 2 min, following which they were incubated for 10 min at 25 °C. Subsequently, the suspension was passed through a 20 µm nylon mesh. The DAPI-stained nuclei were analyzed using a Partec PAII flow cytometer (Partec). The populations of 2 and 4C nuclei were measured on 10,000 nuclei.

The extraction and detection of β-tubulin was conducted by Western blotting. Proteins were extracted from RCs (50) or PCs (50) isolated from caryopses after imbibition for various periods of time in distilled water or KAR_1_ (3 × 10^−9^ M). All samples were ground to a fine powder in liquid nitrogen using a Retsch MM200 laboratory mill ball and homogenized in the lysis buffer containing 62.5 mM Tris–HCl (pH 6.8), 2 % (w/v) SDS, 15 mg/ml DTT and 7 % (v/v) glycerol. After homogenization, the samples were boiled for 10 min and centrifuged for 7 min at 17,000*g*. Samples containing 50 µg protein were loaded per line and separated on 15 % SDS-PAGE gel following Laemmli (1970). Pure bovine brain tubulin (Cytoskeleton, Denver, CO, USA) was loaded as a control in amounts of 10 and 30 ng (molecular mass ~50 kDa). After electrophoresis, the gels were electroblotted onto PVDF membranes (Millipore). Following triple washing in TBST, the blotting membranes were incubated in a blocking solution and probed with the mouse monoclonal anti-β-tubulin antibody (clone KMX-1) (Millipore). The membranes were then washed three times in TBST and probed with peroxidase-conjugated secondary antibody (Millipore). The immunoblots were incubated with a detection solution containing acetate buffer, diaminobenzidine and H_2_O_2_. The data are as immunoblot band visualization and densitometry analysis of immunoblot β-tubulin (ng). The band intensities were determined using the Fiji ImageJ software.

### Determination of ascorbate (AsA) and dehydroascorbate (DHA) contents

The AsA and DHA contents were determined as described by Kampfenkel et al. (1995). Caryopses (25 in three replicates) were incubated either in water or in the solution of 3 × 10^−9^ M KAR_1_ or 10^−5^ M GA_3_ for up to 28 h. At 4 h intervals of imbibition, embryos were isolated from the caryopses. Twenty-five embryos were ground to a fine powder in liquid nitrogen using a Retsch MM200 laboratory mill ball and homogenized for 10 min in 0.8 ml of 6 % (w/v) TCA (fresh weight:TCA, 1:8, w/v). The extracts were centrifuged at 15,600*g* for 10 min, and the resulting supernatants were used for assays. The supernatant (100 µl) or 6 % (w/v) TCA was added to the reaction mixture:

**Table:** 

Compound	AsA		Total AsA	
	Test (ml)	Blank (ml)	Test (ml)	Blank (ml)
0.2 M K-buffer pH 7.4	0.25	0.25	0.25	0.25
10 mM DTT	–	–	0.05	0.05
Incubation: 10 min at 25 °C				
0.5 % (w/v) NEM	–	–	0.05	0.05
ddH_2_O	0.1	0.1	–	–
10 % (w/v) TCA	0.2	0.2	0.2	0.2
44 % (v/v) H_3_PO_4_	0.2	0.2	0.2	0.2
4 % (w/v) 2,2′-dipyridyl	0.2	0.2	0.2	0.2
3 % (w/v) FeCl_3_	0.2	0.2	0.2	0.2
Incubation: 40 min at 42 °C				

The absorbance of the end product was measured at 525 nm using water as a reference. Spectrophotometric analyses were conducted on an UV-VIS spectrophotometer (Thermo Fisher Scientific, Madison, USA). A standard curve was prepared by using the AsA standard. To obtain total AsA, DHA presented in sample was reduced by DTT to AsA. The amount of DHA was calculated as the difference between the total AsA and AsA (before pretreatment with DTT). The results are expressed as µM AsA g^−1^ FW.

### Native-PAGE and ascorbate peroxidase (APX) staining activity

To analyze the APX activity in native-PAGE, total protein was extracted, under non-denaturing conditions, from *A. fatua* L. embryos, 25 in 3 replicates, isolated from caryopses incubated for 0, 12 and 24 h in the presence of KAR_1_ (3 × 10^−9^ M) or GA_3_ (10^−5^ M). All the samples were ground to a fine powder in liquid nitrogen using a Retsch MM200 laboratory mill ball and homogenized in 0.1 M Na-phosphate buffer (pH 7.0) containing 1 mM EDTA, 5 mM ascorbate and 10 % (v/v) glycerol (fresh weight:extraction buffer, 1:5, w/v). The insoluble material was removed by centrifugation at 14,000*g* for 10 min at 4 °C, and the supernatant was used. Samples containing 40 µg protein were loaded onto a 10 % polyacrylamide gel. The protein was separated using native-PAGE at 4 °C and 150 V in the Laemmli (1970) buffer system without SDS. After electrophoresis run to identify APX isoforms, the native gel was incubated for 30 min in 50 mM Na-phosphate buffer (pH 7.0) containing 2 mM ascorbate and for 20 min in 50 mM Na-phosphate buffer (pH 7.0) containing 4 mM ascorbate and 2 mM H_2_O_2_. After rinsing for 1 min with 50 mM Na-phosphate buffer (pH 7.0), the gel was stained with 50 mM Na-phosphate buffer (pH 7.8) containing 28 mM TEMED and 2.5 mM NBT (Mittler and Zilinskas 1993). Colorless bands of APX isoenzymes appeared as the gel was stained blue. Gel images were captured with a G BOX F3 (Syngene, Cambridge, UK) gel documentation system with inversed colors.

### Determination of glutathione reduced form (GSH) and glutathione oxidized form (GSSG) contents

GSH and GSSG form was assayed following Smith (1985). Caryopses (25 in 3 replicates) were incubated either in water or in the solution of 3 × 10^−9^ M KAR_1_ or 10^−5^ M GA_3_ for up to 28 h. At 4 h intervals during caryopsis imbibition, embryos were isolated from the caryopses. Twenty-five embryos were ground to a fine powder in liquid nitrogen using a Retsch MM200 laboratory mill ball. The ground samples were extracted in 1 ml of ice cold 5 % (w/v) sulfosalicylic acid (fresh weight:sulfosalicylic acid, 1:10, w/v) and centrifuged at 10,000*g* for 20 min at 4 °C. A 1 ml aliquot of the supernatant was neutralized by adding 1.5 ml of 0.5 M potassium phosphate buffer (pH 7.5) and used to measure total glutathione (GSH+GSSG). Another 1 ml of the neutralized supernatant was pretreated with 0.2 ml of 2-vinylpyridine for 1.5 h at 25 °C to mask GSH and to allow determination of GSSG alone. Both samples were extracted twice with 5 ml of diethylether. The incubation mixture contained: 0.5 ml of 0.1 M sodium phosphate buffer (pH 7.5) with 5 mM EDTA, 0.2 ml of 5 mM 5,5′-dithiobis-(2-nitrobenzoic acid), 0.1 ml of 2 mM NADPH, 0.1 ml of glutathione reductase type III and 0.1 ml of extract. The change in absorbance at 412 nm was followed at 25 °C. A standard curve was prepared using the GSH standard. The amount of reduced glutathione was calculated as the difference between the total and the oxidized glutathione. The results are expressed as nM GSH g^−1^ FW and as nM GSSG g^−1^ FW.

### Determination of glutathione reductase (GR) activity

Caryopses (25 in five replicates) were incubated either in water or in the solution of 3 × 10^−9^ M KAR_1_ or 10^−5^ M GA_3_ for up to 28 h. At 4 h intervals during caryopsis imbibition, embryos were isolated from caryopses. Twenty-five embryos were ground to a fine powder in liquid nitrogen using a Retsch MM200 laboratory mill ball and homogenized for 10 min in 0.1 M potassium phosphate buffer (pH 7.0) containing 10 mM EDTA and 1 % (w/v) PVP (fresh weight:extraction buffer, 1:10, w/v). The homogenates were centrifuged for 20 min at 15,000*g* at 4 °C. The GR activity was analyzed as described by Esterbauer and Grill (1978) by following the rate of NADPH oxidation at 340 nm for 3 min. The assay mixture contained: 0.1 mM potassium phosphate buffer (pH 7.8), 0.5 mM NADPH, 10 mM oxidized glutathione (GSSG), 10 mM EDTA and 100 µl of enzyme extract. The GR activity was expressed as nM NADPH min^−1^ mg^−1^ protein.

### Protein assay

The protein content in the enzymatic extracts was assayed following Bradford (1976), using bovine serum albumin (BSA) as the standard.

### Statistical analysis

The mean ± standard deviation (SD) of three or five replicates are shown. The means were also analyzed for significance using one-way or two-way analysis of variance, ANOVA (Statistica for Windows v. 10.0, Stat-Soft Inc., Tulsa, OK, USA). Duncan’s multiple range test was used to test for significance of differences (*P* ≤ 0.05) between germination percentage and biochemical assay results of *A. fatua* L. caryopses. Similar results were obtained in two independent experiments.

## Results

### Effects of KAR_1_ and GA_3_ on water uptake and caryopses germination

**Fig. 2:**
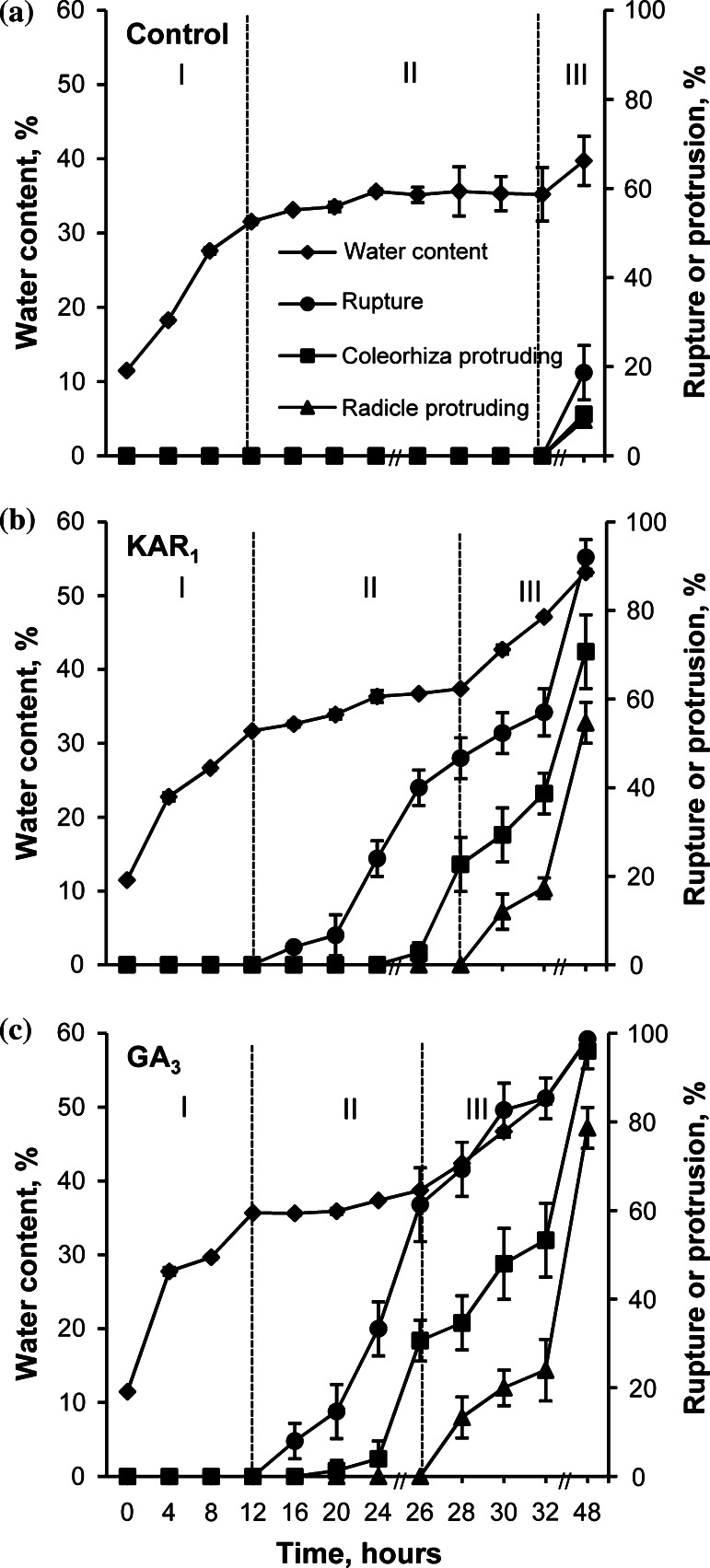
Effects of KAR_1_ or GA_3_ on the water content, coat rupture, coleorhiza protrusion and radicle protrusion in *A. fatua* caryopses during imbibition at 20 °C for various times. *Vertical bars* indicate ± SD (*n* = 3)

Water uptake by dormant caryopses incubated in water or in the presence of KAR_1_ increased up to 12 h of incubation to the same level (Fig. 2a, b). GA_3_ enhanced water uptake to a little higher level (Fig. 2c). The period up to 12 h is considered as phase I of imbibition. At the beginning of phase II, starting from 12 h, the water content slightly increased up to 24 h, both in the caryopses incubated in water (Fig. 2a), in the presence of KAR_1_ or GA_3_ (Fig. 2b, c). Later on, the water content did not change up to 32 h in the untreated and up to 28 or 26 h in treated caryopses. Beginning from 20 or 16 h the percentage of coat rupture increased with incubation time in KAR_1_- or GA_3_-treated caryopses (Figs. 1, 2b, c). KAR_1_ and GA_3_ increased protrusion of coleorhiza through the covering structures, beginning from 28 to 26 h, respectively. Two hours later, at the beginning of phase III, caryopses with coleorhiza protrusion by the radicle started to appear. The increase in water uptake during this phase was associated with a progressive increase in the percentage of coleorhiza and radicle protrusion (germination); after 48 h, ca 60 or 80 % germinated caryopses were observed in the KAR_1_ and GA_3_ treatment, respectively. Phase III was distinct in the treated caryopses only. After 48 h, the water uptake by untreated caryopses slightly increased, and only ca. 20, 10, and 10 % caryopses showed coat rupture, coleorhiza protrusion and radicle protrusion, respectively (Fig. 2a). Prolonged incubation did not increase the percentage of untreated caryopses with radicle protrusion (Table 3).

### Effects of KAR_1_ and GA_3_ on the size of embryo and length of coleorhiza

**Fig. 3:**
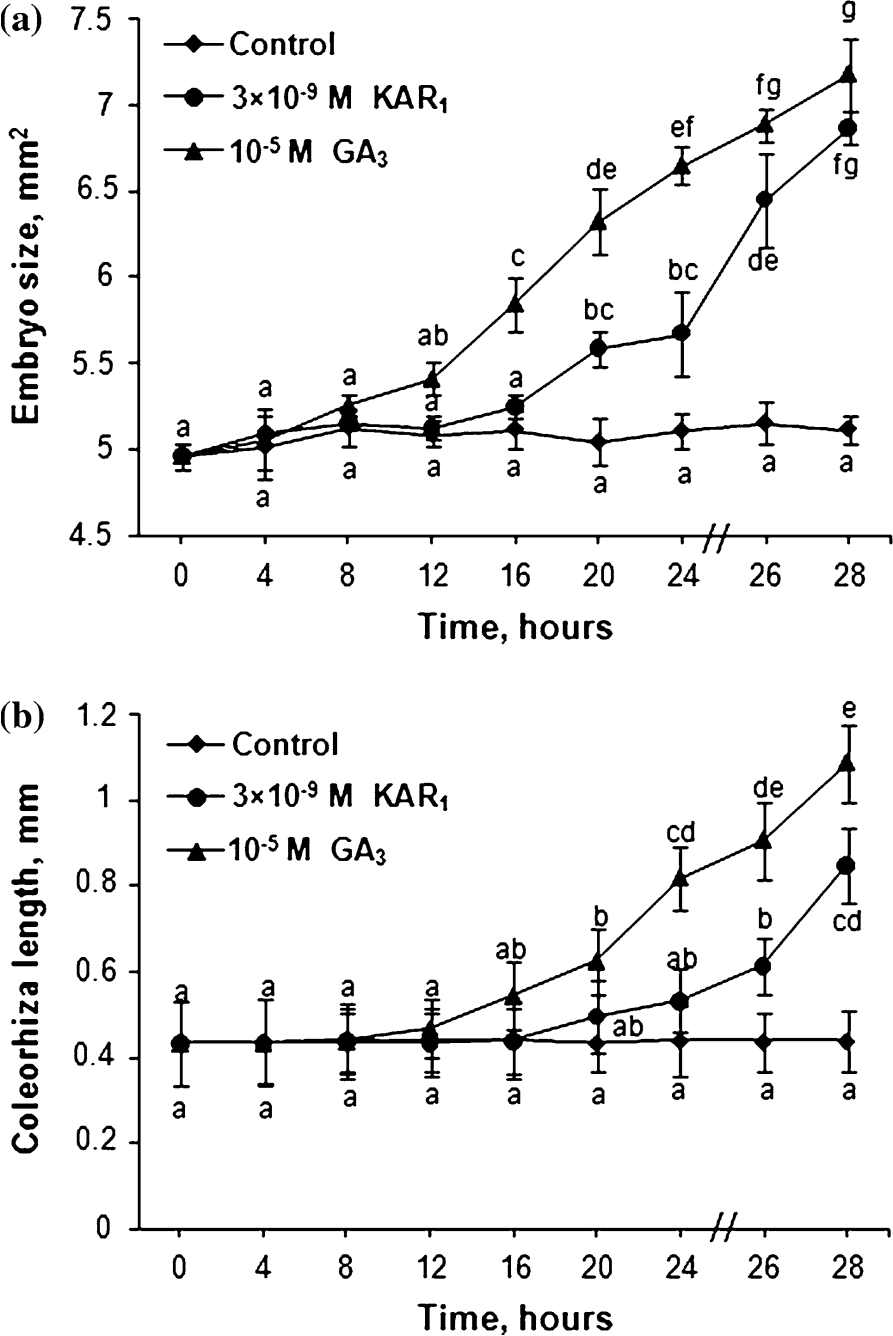
Effects of KAR_1_ or GA_3_ on the embryo size (**a**) and coleorhiza length (**b**) in *A. fatua* caryopses after incubation at 20 °C for various times. *Vertical bars* indicate ± SD. One-way ANOVA with the Duncan’s post hoc test was used to identify significant differences. Means with *different letters* (*a*–*d*) are significantly different (*P* < 0.05, *n* = 3)

Effects of KAR_1_ and GA_3_ on the embryo growth and the length of coleorhiza in caryopses incubated for 28 h were determined. The embryo size in caryopses incubated in water did not change during 28 h incubation (Fig. 3a). KAR_1_ and GA_3_ increased the embryo size beginning from 20 h and from 16 h, respectively; at the end of incubation, the embryos were by 34–40 % larger than the embryos of caryopses incubated in water. The length of coleorhiza did not change during the whole period of caryopsis incubation in water; however, KAR_1_ and GA_3_ increased the length beginning from 26 and 20 h of incubation, respectively (Fig. 3b). Incubation for 28 h in the presence of KAR_1_ or GA_3_ resulted in the coleorhiza being by 90 or 150 % longer, respectively, compared to the control.

### Effects of KAR_1_ and GA_3_ on contents of ABA, PA, DPA, neoPA and 7′OHABA

**Fig. 4:**
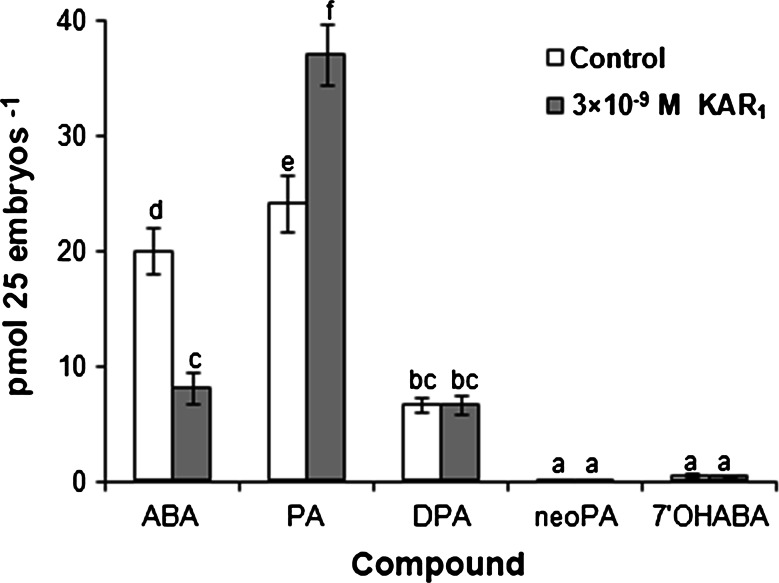
Effects of KAR_1_ on ABA, PA, DPA, neoPA and 7′OHABA content in embryos isolated from *A. fatua* caryopses incubated at 20 °C for 20 h. *Vertical bars* indicate ± SD. Two-way ANOVA with the Duncan’s post hoc test was used to identify significant differences. Means with *different letters* (*a*–*f*) are significantly different (*P* < 0.05, *n* = 3)

Effects of KAR_1_ on contents of ABA and its metabolites: PA, DPA, neoPA and 7′OHABA in embryos isolated from caryopses incubated for 20 h were determined. The PA level in embryos from untreated caryopses was somewhat higher than the level of ABA (Fig. 4). The DPA concentration was much lower, and the contents of neoPA and 7′OHABA were very low. KAR_1_ decreased the ABA content by 60 % and increased the PA content by 55 %. The contents of DPA, neoPA and 7′OHABA were similar in embryos from untreated and treated caryopses.

### Effects of KAR_1_ and GA_3_ on nuclear DNA content and β-tubulin accumulation

Effects of KAR_1_ on the nuclear DNA content in radicle with coleorhiza (RC) and plumule with coleoptile (PC) were determined. Most cells in RC tips of dry caryopses contained nuclei with 2C values, indicating that the majority of cells were arrested at phase G_1_ (Table 1). Imbibition in water from 24 up to 28 h did not change the percentage of nuclei in 2C DNA, at phase S and at 4C in RC. Both KAR_1_ and GA_3_ decreased the number of nuclei with 2C DNA in RC after 24 h, and more markedly after 28 h, of imbibition. After 24 h the number of nuclei was by 15 or 30 % lower in RC from KAR_1_ and GA_3_ treatment, respectively, than in RC from untreated caryopses, and after 28 h a 30 and 50 % reduction was observed in the KAR_1_ and GA_3_ treatment, respectively. During the whole period of imbibition, the number of nuclei in phase S was higher in RC from KAR_1_- and GA_3_-treated caryopses than from untreated ones. The number of nuclei with 4C DNA in RC from KAR_1_- and GA_3_-treated caryopses increased progressively with incubation time: after 24 h it was 1.5 and 3 times higher in RC of the KAR_1_- and GA_3_-treated caryopses, respectively, than in the untreated ones, and after 28 h it was ca. 3 and 6 times higher. When caryopses were imbibed in water, the numbers of nuclei of 2C, S and 4C DNA in PC did not change during the whole period of imbibition. KAR_1_ induced a decrease in the number of 2C in PC only after 28 h, the effect of GA_3_ being visible after 26 and 28 h (Table 1). S DNA nuclei in PC was increased after 26 and 28 h by KAR_1_ and during the whole period of imbibition by GA_3_. Both KAR_1_ and GA_3_ increased the number of 4C DNA nuclei in PC, after 28 and 26–28 h, respectively.

**Table 1:** Effects of KAR_1_ or GA_3_ on the nuclear DNA content and 4C/2C ratio in *A. fatua* radicle with coleorhiza and plumule with coleoptile after caryopsis incubation at 20 °C for 24, 26 or 28 h

Imbibition (h)	Number of nuclei (%)						4C/2C (10^−2^)	
	2C		S			4C		
Radicle with coleorhiza								
	Control	KAR_1_	Control	KAR_1_	Control	KAR_1_	Control	KAR_1_
0	92.8 ± 1.0c	-	3.7 ± 0.8a	-	3.5 ± 0.4a	-	3.8 ± 0.5a	-
24	89.8 ± 0.6c	77.3 ± 1.5b	5.9 ± 0.4b	16.0 ± 1.7cd	4.4 ± 0.4a	6.7 ± 0.4b	4.9 ± 0.5ab	8.6 ± 0.5c
26	88.3 ± 1.1c	72.2 ± 1.9b	6.8 ± 0.6b	18.4 ± 1.9d	4.9 ± 0.6ab	9.4 ± 1.5c	5.5 ± 0.7b	13.0 ± 2.3d
28	88.1 ± 1.4c	63.0 ± 1.1a	6.9 ± 0.6b	20.1 ± 2.3d	5.0 ± 0.9ab	16.7 ± 1.2d	5.7 ± 1.1b	26.5 ± 1.5e
	Control	GA_3_	Control	GA_3_	Control	GA_3_	Control	GA_3_
0	92.8 ± 1.0d	-	3.7 ± 0.8a	-	3.5 ± 0.4a	-	3.8 ± 0.5a	-
24	89.8 ± 0.6d	64.6 ± 0.6c	5.9 ± 0.4b	22.6 ± 0.3c	4.4 ± 0.4ab	12.8 ± 0.5c	4.9 ± 0.5a	19.8 ± 0.9c
26	88.3 ± 1.1d	54.3 ± 0.6b	6.8 ± 0.6b	25.3 ± 0.7d	4.9 ± 0.6b	20.4 ± 0.3d	5.5 ± 0.7ab	37.5 ± 0.5d
28	88.1 ± 1.4d	45.1 ± 0.8a	6.9 ± 0.6b	24.3 ± 1.2cd	5.0 ± 0.9b	30.6 ± 1.0e	5.7 ± 1.1ab	67.8 ± 2.4e
Plumule with coleoptile								
	Control	KAR_1_	Control	KAR_1_	Control	KAR_1_	Control	KAR_1_
0	86.9 ± 1.8b	-	8.2 ± 1.1ab	-	5.0 ± 0.7a	-	5.7 ± 1.0a	-
24	88.5 ± 1.7b	86.7 ± 1.0b	7.0 ± 0.7a	8.2 ± 0.6ab	4.6 ± 1.1a	5.1 ± 0.5a	5.3 ± 1.3a	5.8 ± 0.7a
26	87.1 ± 0.7b	84.9 ± 1.5b	7.4 ± 0.5a	9.5 ± 0.5b	5.3 ± 0.5a	5.6 ± 1.3a	6.1 ± 0.6a	6.6 ± 1.7a
28	86.8 ± 0.5b	74.7 ± 0.5a	7.3 ± 0.6a	12.9 ± 0.8c	5.9 ± 0.6a	12.4 ± 0.4b	6.8 ± 0.7a	16.6 ± 0.5b
	Control	GA_3_	Control	GA_3_	Control	GA_3_	Control	GA_3_
0	86.9 ± 1.8c	-	8.2 ± 1.1b	-	5.0 ± 0.7a	-	5.7 ± 1.0ab	-
24	88.5 ± 1.7c	85.3 ± 0.8c	7.0 ± 0.7a	8.4 ± 0.5b	4.6 ± 1.1a	5.0 ± 1.1a	5.3 ± 1.3a	7.0 ± 1.4b
26	87.1 ± 0.7c	77.7 ± 1.1b	7.4 ± 0.5ab	10.7 ± 1.7c	5.3 ± 0.5a	11.5 ± 1.0b	6.1 ± 0.6ab	14.8 ± 1.2c
28	86.8 ± 0.5c	67.3 ± 1.3a	7.3 ± 0.6ab	16.2 ± 0.9d	5.9 ± 0.6a	15.6 ± 0.7c	6.8 ± 0.7b	21.1 ± 1.3d

The 28-h-long KAR_1_ treatment resulted in ca. 23% of the caryopses with the protruding coleorhiza. The 28-h-long GA_3_ treatment resulted in ca. 28% of the caryopses with the protruding coleorhiza and 16 % of the caryopses with radicle penetrating the coleorhiza. Two-way ANOVA with the Duncan’s post hoc test was used to identify significant differences. Means with different letters (a–d) are significantlv different (*P* *<* 0.05, *n* = 5)

In dry caryopses, the 4C/2C ratio was very low in RC, and did not change during the whole period of caryopsis imbibition in water. It increased for RC by KAR_1_ and GA_3_ progressively with time of incubation. The ratio was 1.8 or 4 and 5 or 12 times higher in the caryopses imbibed for 24 and 28 h in the presence of KAR_1_ or GA_3_, respectively, than in caryopses imbibed in water. In PC, the ratio was similar for dry and imbibed caryopses in water for up to 28 h or in the KAR_1_ solution for up to 26 h. After 28 h KAR_1_ increased the 4C/2C ratio in PC 2.5 times compared with the control. GA_3_ increased the 4C/2C ratio in PC during the whole period of imbibition; after 24 h 1.3-fold and after 28 h a 3-fold increase was found, compared to the control.

Western blot analysis was used to detect β-tubulin in RC and PC of dry caryopses and those imbibed in water, the KAR_1_ or GA_3_ solutions for up to 28 h. β-Tubulin was not detected in RC from dry caryopses and also from caryopses incubated in water for 24 h (Table 2). The exposure to KAR_1_ or GA_3_ during imbibition resulted in β-tubulin being detected in RC just after 24 h. Prolongation of incubation in the presence of both compounds for up to 28 h progressively increased the intensity of the signal. The intensity of this signal was higher than the intensity of the signal for RC of caryopses imbibed in water. Data from the densitometric analysis are in good agreement with the results obtained with Western blotting. The analyses showed the absence of β-tubulin in RC from dry caryopses and caryopses incubated in water for 24 h. The β-tubulin contents after 26 and 28 h imbibition in water were similar. KAR_1_ and GA_3_ increased the β-tubulin content after all periods of incubation, compared with the controls, the highest effect being visible after 28 h when the increase was ca. 7- and 15-fold, respectively. A low intensity β-tubulin signal was detected in PC only when caryopses were incubated in the presence of KAR_1_ or GA_3_ for 26 and 28 h. The densitometric analysis allowed to measure the β-tubulin content in PC only when caryopses were treated with KAR_1_ or GA_3_ for 26 and 28 h. After 28 h incubation, the β-tubulin content was ca. 5 times lower in PC than in RC from KAR_1_- or GA_3_-treated caryopses.

**Table 2:**
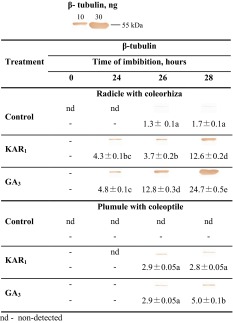
Effects of KAR_1_ or GA_3_ on the β-tubulin content in *A. fatua* radicle with coleorhiza and plumule with coleoptile after caryopsis incubation at 20 °C for 24, 26 or 28 h

Data are presented as immunoblott band visualization and densitometry analysis of immunoblott β-tubulin (ng). Positions of tubulin control in amounts of 10 and 30 ng (molecular mass ~50 kDa) are shown above the table. The 28-h-long KAR_1_ treatment resulted in ca. 23 % of the caryopses with the protruding coleorhiza. The 28-h-long GA_3_ treatment resulted in ca. 28 % of the caryopses with the protruding coleorhiza and 16 % of the caryopses with radicle penetrating the coleorhiza. Two-way ANOVA with the Duncan’s post hoc test was used to identify significant differences. Means with different letters (a–d) are significantly different (*P* < 0.05, *n* = 5)

*nd* not detected

### Effects of KAR_1_ and GA_3_ on caryopsis germination in the absence and presence of AsA, GSH or lycorine

**Fig. 5:**
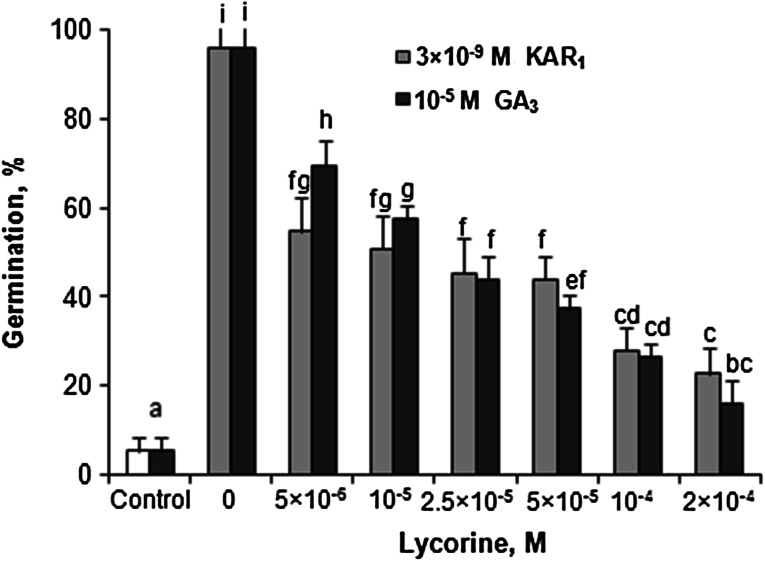
Effects of KAR_1_ or GA_3_ in the absence or presence of lycorine on the germination of *A. fatua* caryopses at 20 °C for 5 days. *Vertical* *bars* indicate ± SD. One-way ANOVA with the Duncan’s post hoc test was used to identify significant differences. Means with *different letters* (*a*–*f*) are significantly different (*P* < 0.05, *n* = 3)

Most of the dormant *A. fatua* caryopses were not able to germinate (Table 3). KAR_1_ and GA_3_ markedly increased germination after 2 days and resulted in almost complete germination after 5 days. ASA and GSH did not affect germination. Both compounds decreased the germination percentage only when applied simultaneously with KAR_1_ or GA_3_ during 2 days. Lycorine, an inhibitor of AsA synthesis, lowered the stimulatory effect of KAR_1_ and GA_3_ (Fig. 5) when used at all concentrations tested. The highest inhibition was noted when lycorine was applied at concentrations higher than 5 × 10^−5^ M simultaneously with KAR_1_ or GA_3_; only about 15–25 % of caryopses germinated.

**Table 3:** Effects of KAR_1_ or GA_3_ in the absence or presence of AsA or GSH on the germination of *A. fatua* caryopses at 20 °C for 2 or 5 days

Compound	Germination (%)	
	Time (days)	
	2	5
H_2_O	4.0 ± 0.0a	9.3 ± 2.3a
KAR_1_, 3 × 10^−9^ M	57.3 ± 4.6d	92.0 ± 4.0f
GA_3_, 10^−5^ M	78.7 ± 2.3e	97.3 ± 2.3f
AsA, 10^−3^ M	6.7 ± 4.6a	8.0 ± 4.0a
AsA+KAR_1_	45.3 ± 2.3c	94.7 ± 2.3f
AsA+GA_3_	28.0 ± 6.9b	96.0 ± 6.9f
GSH, 10^−3^ M	4.0 ± 4.0a	8.0 ± 4.0a
GSH+KAR_1_	30.7 ± 4.6b	89.3 ± 4.6f
GSH+GA_3_	56.0 ± 6.9d	93.3 ± 6.1f

Two-way ANOVA with the Duncan’s post hoc test was used to identify significant differences. Means with different letters (a–e) are significantly different (*P* < 0.05, *n * = 3)

### Effects of KAR_1_ and GA_3_ on AsA and DHA contents

**Fig. 6:**
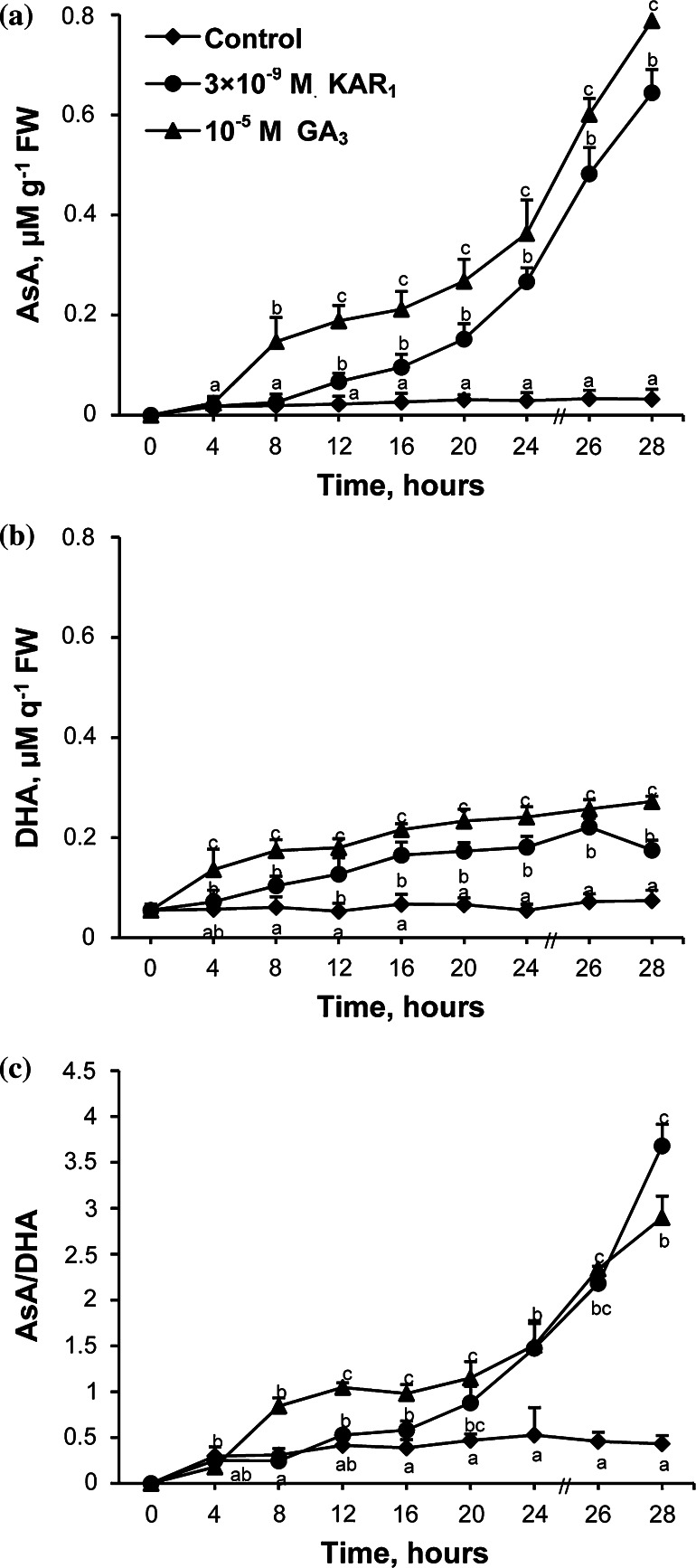
Effects of KAR_1_ or GA_3_ on the AsA (**a**), DHA (**b**) level and AsA/DHA ratio (**c**) in *A. fatua* embryos after caryopses incubation at 20 °C for various times. The 28-h-long KAR_1_ treatment resulted in ca. 24 % of the caryopses with the protruding coleorhiza. The 28-h-long GA_3_ treatment resulted in ca. 35 % of the caryopses with the protruding coleorhiza and 16 % of the caryopses with radicle penetrating the coleorhiza. *Vertical bars* indicate ± SD. One-way ANOVA with the Duncan’s post hoc test was used to identify significant differences. Means with *different letters* (*a*–*g*) are significantly different (*P* < 0.05, *n* = 3)

AsA and DHA contents were determined in embryos isolated from caryopses after imbibition in the presence of KAR_1_ or GA_3_ for various periods. AsA was not detected in embryos from dry caryopses, and the level of the compound did not change during whole period of imbibition in water (Fig. 6a). KAR_1_ and GA_3_ increased the AsA content in embryos from 12 to 8 h, respectively, to the end of imbibition. The highest stimulatory effect was found after 28 h; the AsA content increased 20 to 25 times, compared to the control. Embryos from dry caryopses showed levels of DHA similar to those in embryos from caryopses incubated in water for up to 28 h (Fig. 6b). KAR_1_ and GA_3_ increased the DHA content from 8 and 4 h, respectively, to the end of imbibition. After 28 h of incubation, the DHA content increased ca. 3 or 4 times as a result of the KAR_1_ or GA_3_ treatment, respectively. The AsA/DHA ratio did not change during imbibition of caryopses in water (Fig. 6c). Incubation in the presence of KAR_1_ or GA_3_ increased the ratio gradually from 20 or 8 h, respectively, until the end of imbibition. At the end of the incubation period, the ratio was 9 or 7 times higher in the treated than in the embryos from untreated caryopses.

### Effects of KAR_1_ and GA_3_ on electrophoretic isoenzyme pattern of APX

APX was found to be represented by four isoforms (Table 4). All the isoforms were found in embryos from dry caryopses and also in those from caryopses incubated for 12 and 24 h in water, in the presence of KAR_1_ or GA_3_. The KAR_1_ and GA_3_ treatment resulted in the appearance of an additional isoform, APX-3, after 12 and 24 h.

**Table 4:**
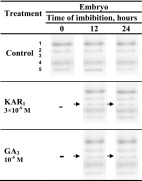
Effects of KAR_1_ or GA_3_ on APX isoenzymes activities in *A. fatua* embryos after caryopsis incubation at 20 °C for 12 or 24 h

### Effects of KAR_1_ and GA_3_ on GSSG and GSH contents and GR activity

**Fig. 7:**
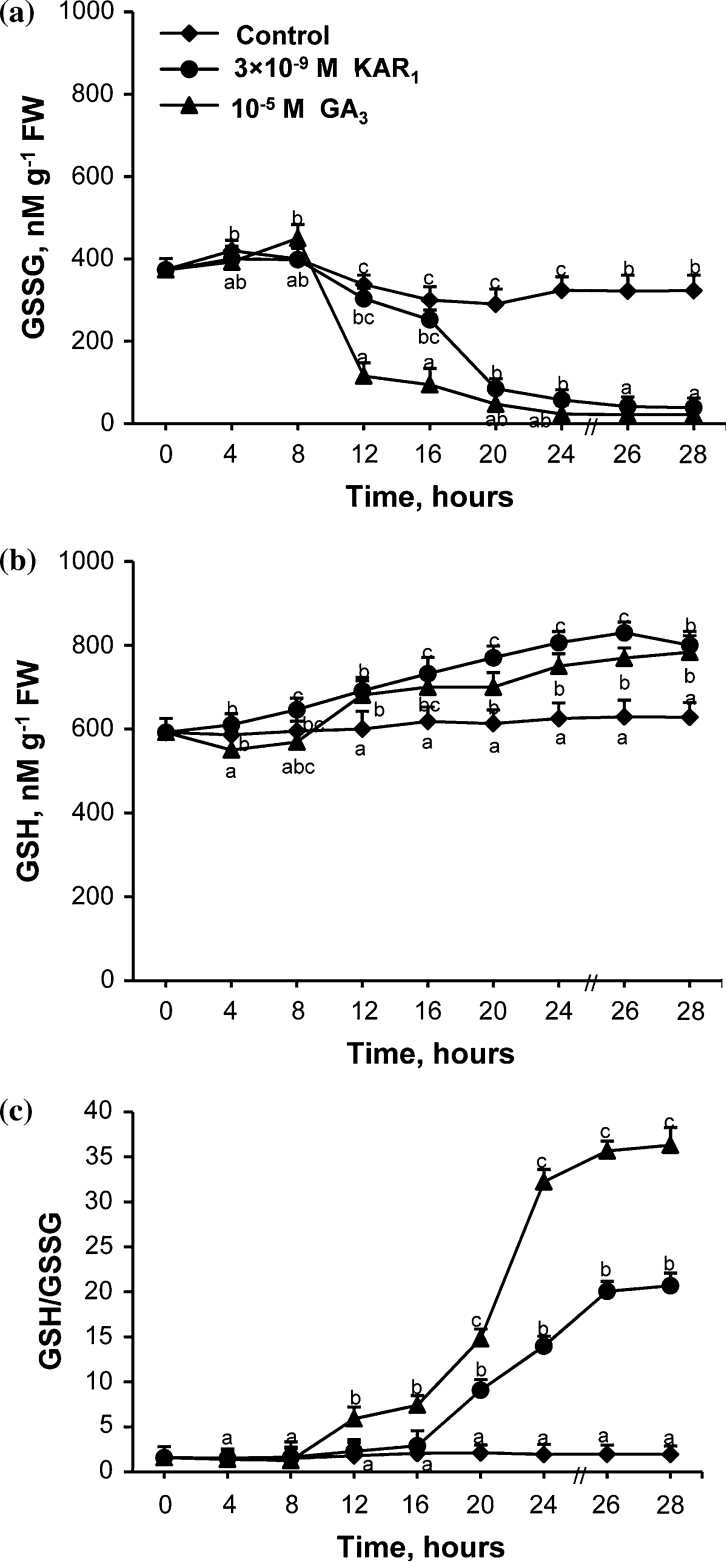
Effects of KAR_1_ or GA_3_ on the level of GSSG (**a**), GSH form (**b**) and GSH/GSSG ratio (**c**) in *A. fatua* embryos after caryopses incubation at 20 °C for various times. The 28-h-long KAR_1_ treatment resulted in ca. 25 % of the caryopses with the protruding coleorhiza. The 28-h-long GA_3_ treatment resulted in ca. 36 % of the caryopses with the protruding coleorhiza and 17 % of the caryopses with radicle penetrating the coleorhiza. *Vertical bars* indicate ± SD. One-way ANOVA with the Duncan’s post hoc test was used to identify significant differences. Means with *different letters* (*a*–*g*) are significantly different (*P* < 0.05, *n* = 3)

**Fig. 8:**
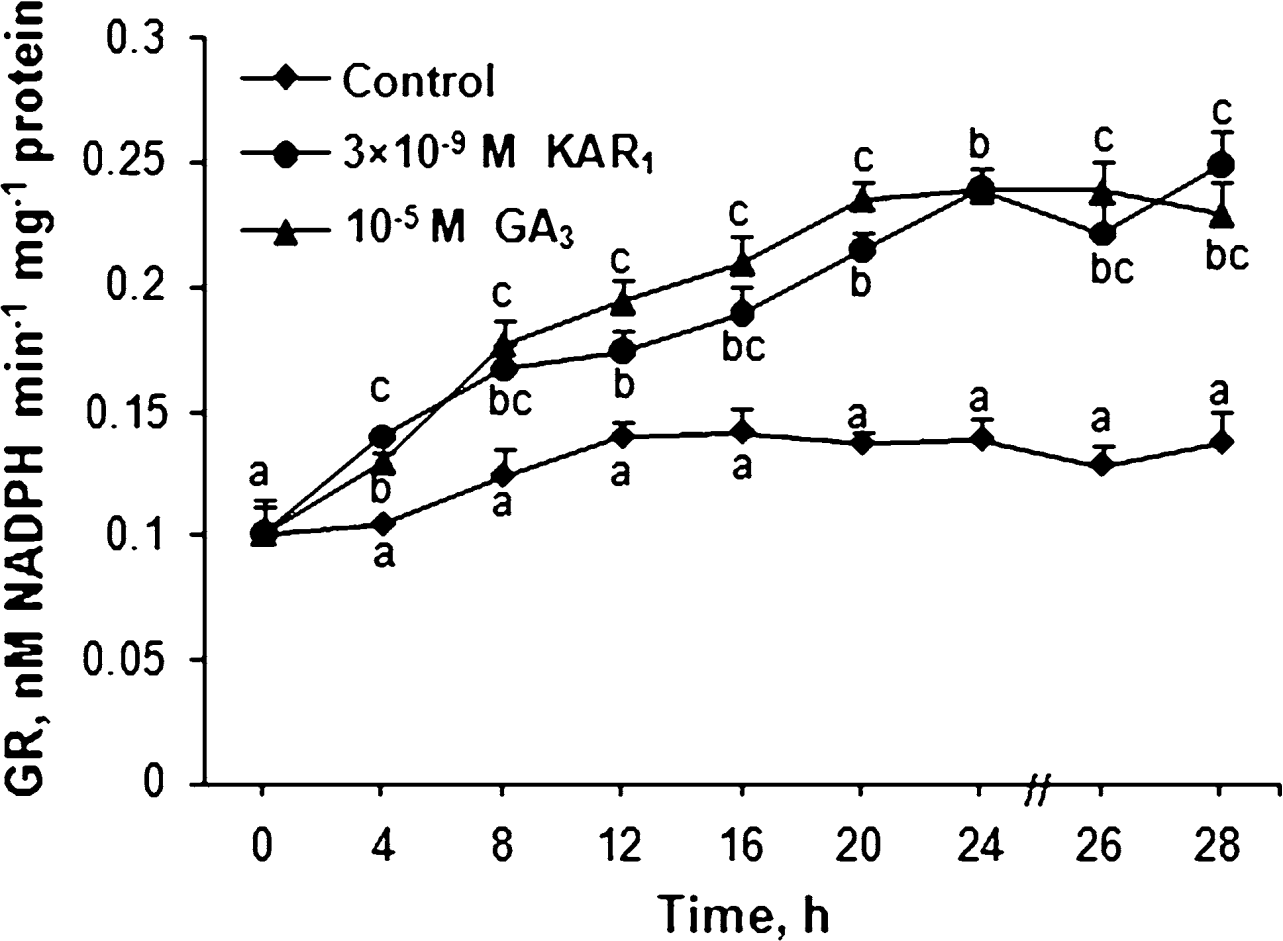
Effects of KAR_1_ or GA_3_ on the GR activity in *A. fatua* embryos after caryopses incubation at 20 °C for various times. The 28-h-long KAR_1_ treatment resulted in ca. 28 % of the caryopses with the protruding coleorhiza. The 28-h-long GA_3_ treatment resulted in ca. 32 % of the caryopses with the protruding coleorhiza and 15 % of the caryopses with radicle penetrating the coleorhiza. *Vertical bars* indicate ± SD. One-way ANOVA with the Duncan’s post hoc test was used to identify significant differences. Means with *different letters* (*a*–*h*) are significantly different (*P* < 0.05, *n* = 5)

The GSSG and GSH contents in embryos from caryopses incubated in the presence of KAR_1_ and GA_3_ for various periods were measured. Embryos from dry caryopses showed a lower content of GSSG than of GSH (Fig. 7). The GSSG content decreased somewhat up to 20 h and later did not change up to the end of imbibition of caryopses in water (Fig. 7a). KAR_1_ decreased the GSSG content in embryos in comparison to the control between 20 and 28 h of imbibition, the GA_3_ effect being visible between 12 and 28 h. KAR_1_ and GA_3_ progressively increased the GSH content (Fig. 7b) from 12 h up to the end of incubation. After 28 h, both KAR_1_ and GA_3_ decreased the content of GSSG by ca. 90 % and increased the GSH content by 25 %. The GSH/GSSH ratio was similar in embryos from dry caryopses and those caryopses imbibed in water for 28 h (Fig. 7c). KAR_1_ or GA_3_ progressively increased the ratio starting from 20 or 12 h of incubation, respectively. After 28 h, the ratio in treated caryopses with KAR_1_ or GA_3_ was by ca. 11 or 19 times higher, respectively, than in the untreated ones. The GR activity in embryos from caryopses incubated in water or in the presence of KAR_1_ or GA was also determined. The GR activity in embryos from the caryopses imbibed in water increased up to 12 h; later on, up to 28 h it remained constant (Fig. 8). Both KAR_1_ and GA_3_ enhanced the GR activity from 4 h. After 28 h, the GR activity was increased by 80 and 70 % by KAR_1_ and GA_3_, respectively.

## Discussion

### Water uptake and germination

Dormant and non-dormant viable dry *orthodox* seeds take up water in two phases; in phase I, the water uptake is rapid, the water content in phase II being constant or increasing only slightly (Bewley et al. 2013). KAR_1_ and GA_3_ slightly increased the rapidity of water uptake by *A. fatua* caryopses during phase I (Fig. 2). During phase II, the water uptake was similar in untreated and treated caryopses. However, phase II was longer in the untreated caryopses. Earlier studies showed that non-dormant *A. fatua* caryopses absorbed water more rapidly than dormant ones (Hou et al. 1997). Both KAR_1_ and GA_3_ stimulated germination of dormant *A. fatua* caryopses (Figs. 1, 2; Table 3) determined, like in previous experiments (Kępczyński and Van Staden 2012), as radicle protrusion through coleorhiza. The radicle protrusion was preceded by several events beginning in phase II. At first, KAR_1_ and GA_3_ stimulated coat rupture (Fig. 2), which was associated with increasing size of embryos (Fig. 3a). Somewhat later, KAR_1_ as well as GA_3_ induced elongation of coleorhiza resulting in the coleorhiza springing up. Coleorhiza elongation stimulated by these regulators is associated with accumulation of O_2_^•−^ and H_2_O_2_ on the coleorhiza, particularly on the surface surrounding the radicle (Cembrowska-Lech et al. 2015). It was previously demonstrated that O_2_^•−^ generated in the radicle/hypocotyl region of *Lepidium sativum* seeds is required for embryo expansion (Oracz et al. 2012).

KAR_1_- and GA_3_-treated *A. fatua* caryopses (Fig. 2), similar to non-dormant seeds of many species (Bewley et al. 2013), can enter phase III associated with increase of water uptake and with completion of germination (radicle protrusion). The transition from phase II to III, due to KAR_1_ or GA_3_ was associated with a further increase in the rupture of coat, coleorhiza protrusion and finally in radicle protrusion through coleorhiza. Coleorhiza elongation was observed to cease prior to root emergence in non-dormant barley caryopses, but not in dormant ones (Barrero et al. 2009). It is assumed that the data presented show KAR_1_ and also GA_3_ to promote various effects: water uptake, expansion of embryos, rupture of coat,  increase of coleorhiza length, coleorhiza protrusion and penetration of coleorhiza by radicle in *A. fatua* caryopses. This stimulatory effect of KAR_1_ and GA_3_ on dormancy release and germination of *A. fatua* was associated with increasing accumulation of H_2_O_2_ and particularly O_2_^•−^ in embryos (Cembrowska-Lech et al. 2015) just before their extension and coat rupture (Figs. 2, 3). That apoplastic O_2_^•−^ production is required for embryo extension growth and endosperm rupture, two observable effects, during germination of non-dormant *Lepidium sativum* seeds was previously demonstrated as well (Oracz et al. 2012). Barrero et al. (2009) proposed that coleorhiza plays a major role in causing dormancy of barley caryopses by acting as a barrier to root emergence. In view of this idea and the results obtained, KAR_1_ and GA_3_ may be considered as dormancy breaking factors in *A. fatua* caryopses.

### ABA metabolism

The level of ABA, known to play a central role in the establishment and maintenance of seed dormancy (Rodríguez-Gacio et al. 2009) and balance of ABA/GAs levels and the sensitivity to these hormones  are mainly  responsible for the equilibrium between dormancy and germination (Finkelstein et al. 2008; Rodríguez-Gacio et al. 2009). The content of ABA was higher in embryos isolated from dry dormant caryopses of *A. fatua* (Cembrowska-Lech et al. 2015) or barley (Millar et al. 2006; Bahin et al. 2011) than from those imbibed in water. KAR_1_ and GA_3_, when present during imbibition of caryopses, decreased the ABA level in embryos in phase II (Cembrowska-Lech et al. 2015; Fig. 4). The reduction of ABA (Cembrowska-Lech et al. 2015) preceded the coleorhiza protrusion and radicle penetration of coleorhiza by radicle (Fig. 2). Thus, germination of both KAR_1_- and GA_3_-treated *A. fatua* caryopses similarly as after-ripened barley caryopses (Millar et al. 2006) was associated with a previous reduction of the ABA content. Interestingly, KAR_1_ was also able to stimulate germination of dormant *Arabidopsisthaliana* seeds without changing the ABA level (Nelson et al. 2009). Therefore, the mechanism of germination induction in dormant seeds controlled by KAR_1_ may be plant species-specific. The decrease of the ABA content in *A. fatua* embryos by KAR_1_ was connected with an increased PA content (Fig. 4). Similarly, a decline in ABA in barley grains was associated with an increase of PA (Jacobsen et al. 2002). It is commonly accepted that catabolism of ABA is associated with its hydroxylation and conjugation (Rodríguez-Gacio et al. 2009). The reduction of the ABA content in *A. fatua* embryos corresponds with the increase of PA caused by KAR_1_, which suggests that removal of ABA is associated mainly with its hydroxylation. ABA hydroxylation by ABA 8′-hydroxylase is thought to be the predominant ABA deactivation pathway in many physiological processes. 8′-Hydroxy ABA isomerizes spontaneously to phaseic acid and can be further metabolized to DPA (Rodríguez-Gacio et al. 2009). The ABA 8′ hydroxylase gene (*HvABA 8′OH*-*1*) was considered as key gene controlling ABA level and primary dormancy in barley (Millar et al. 2006). Taking into account both the evidence referred to above and the data presented in this work, KAR_1_ can be considered as an important regulator controlling dormancy of *A. fatua* caryopses via ABA hydroxylation. Catabolism of ABA could take place in coleorhiza of *A.* *fatua*, as shown for barley by Millar et al. (2006). Similar levels of DPA, neoPA and 7′OHABA in embryos from untreated and KAR_1_-treated caryopses (Fig. 4) may suggest the lack of KAR_1_ effect on catabolism of PA and ABA hydroxylation in positions C-7′ and C-9′.

### Cell cycle activation

Most cells in RC and PC from dry dormant *A. fatua* caryopses were arrested in phase G_1_ of the cell cycle (Table 1). Likewise, the majority of radicle tip nuclei of dry dormant barley contain cells in G_1_ (Gendreau et al. 2012). Dormant *A. fatua* caryopses did not germinate, and the percentage of nuclei in G_1_, S and G_2_ and cell cycle activity (G_2_/G_1_) did not change in RC during imbibition of dormant *A. fatua* caryopses up to 28 h. Likewise, the DNA synthesis was reported earlier not to have increased during imbibition of *A. fatua* dormant caryopses (Elder and Osborne 1993). KAR_1_ and GA_3_, which stimulated germination of *A. fatua* dormant caryopses, decreased the number of cells in phase G_1_ and increased it in phases S and G_2_ in RC during imbibition. Changes in the number of nuclei in RC of *A. fatua* were probably associated only with the radicle, since no cell division was previously found in barley coleorhiza (Barrero et al. 2009). The stimulatory effect of KAR_1_ on the cell cycle initiation appeared after 24 h (Table 1), 4 h prior to coleorhiza protrusion through the covering structures and 6 h before radicle protrusion (Fig. 2); the G_2_/G_1_ ratio in RC was increased ca. 2 times, compared to the untreated (Table 1). Similarly, the cell cycle initiation by GA_3_ occurred, after 24 h, 2 h before coleorhiza protrusion and 4 h before radicle protrusion; the G_2_/G_1_ ratio was increased 4 times (Table 1; Fig. 2). Thus, the induction of release from dormancy and germination of *A. fatua* caryopses by KAR_1_ and also by GA_3_ involves initiation of the cell cycle in radicle before germination. The G_2_/G_1_ ratio in RC was progressively increased by KAR_1_ and after 28 h, when coleorhiza began to protrude (23 %), was 5 times higher than in the control. The increase of coleorhiza (28 %) and radicle (16 %) protrusion by GA_3_ after 28 h was associated with a 12-fold increase of the G_2_/G_1_ ratio. In non-dormant barley grains able to germinate (Gendreau et al. 2008), the cell cycle was initiated in radicle tip before the radicle protrusion through the covering structures (seed coat+pericarp and glumelle). In polysomic species, i.e. *Lepidium sativum*, it was demonstrated that endoreduplication was associated with cell extension radicle/hypocotyl region prior to germination of non-dormant *Lepidium sativum* seeds (Oracz et al. 2012). An experiment with dormant *Lycopersicumesculentum* seeds showed cells to move into phase G_2_ if dormancy was broken (de Castro et al. 2001). Expression of barley grain dormancy at 30 °C was shown to be associated with blocking of the nuclei in phase S (Gendreau et al. 2012). Up to 28 h, KAR_1_ did not affect coleoptile elongation (which emerges after radicle; not shown); however, after 28 h, it induced increase in PC cells in both phases, S and G_2_, and also enhanced the G_2_/G_1_ ratio (Table 1). So, KAR_1_ allows to initiate the cell cycle activity before coleoptile growth starts. Likewise, GA_3_ initiates cell cycle activity before emergence of coleoptile. Thus, the effect of these regulators on further growth of coleoptile includes preparation to cell division. However, previously it was suggested that coleoptile growth is mainly associated with cell extension (Magneschi and Perata 2009). The effect of KAR_1_ on the G_2_/G_1_ ratio was observed later in PC than in RC, indicating that stimulatory effect of this regulator occurs at different times of incubation, depending on the organ.

β-Tubulin, a component of the microtubular cytoskeleton, is known to be required for the cell cycle to proceed. The present results show that RC from dry dormant caryopses and also from those imbibed in water for 24 h did not have β-tubulin; densitometry allowed to detect its low level only after prolonged imbibition (Table 2). β-Tubulin  appeared in RC from KAR_1_- or GA_3_-treated caryopses when cells started to move into phase G_2_. Data from densitometry analyses may suggest that β-tubulin precedes or coincides with DNA replication induced by KAR_1_ or GA_3_ (Table 1). Previous studies showed that β-tubulin accumulation preceded the onset of DNA replication during germination of cabbage seeds (Górnik et al. 1997) or that the two processes can occur simultaneously, as was the case during germination of sugarbeet seeds (Śliwińska et al. 1999).

However, in the case of PC of KAR_1_-treated caryopses, β-tubulin was measurable by densitometry after 26 h thus before transition from G_1_ to G_2_. So, β-tubulin accumulation in PC preceded DNA replication. When caryopses were treated with GA_3_, β-tubulin accumulation coincided with DNA replication. Probably, the β- tubulin synthesis and DNA replication in RC and PC (Tables 1, 2) are possible because the ABA level was decreased by KAR_1_ or GA_3_ (Fig. 4; Cembrowska-Lech et al. 2015) prior to those events. The role of ABA in regulating the cell cycle was shown in other studies as well. A sharp decrease in the ABA content in barley embryo from non-dormant grains correlated with an increase in the cell cycle activity (Gendreau et al. 2008). ABA decreased the level of β-tubulin and the number of cells in phase G_2_ and inhibited germination of coffee seeds (Da Silva et al. 2008). However, it has been recently suggested that the cell cycle regulation during incubation of dormant barley grains is only partially by the metabolism of ABA (Gendreau et al. 2012).

### AsA and DHA contents

It was found earlier that induction of germination in dormant *A. fatua* caryopses by KAR_1_ and GA_3_ was associated with an increase in both the content of ROS, H_2_O_2_ and O_2_^•−^, and activities of SOD and CAT in *A. fatua* embryos to control the ROS–antioxidant status (Cembrowska-Lech et al. 2015). It was postulated that, among ROS, only H_2_O_2_ seems to play a major role (Li et al. 2010), and the ascorbate–glutathione cycle was indicated as mainly responsible for H_2_O_2_ scavenging (Liu et al. 2010). In contrast to embryos of dry dormant barley grains (Bahin et al. 2011), embryos of dry dormant *A. fatua* (Fig. 6a) are devoid of AsA. The AsA and DHA contents in embryos did not change during the whole period of imbibition of dormant *A. fatua* caryopses (Fig. 6a, b ). During imbibition of non-dormant rice seeds, the AsA content was observed to increase, and it was suggested that AsA biosynthesis and APX activity are crucial to ensure seed germinability (Ye et al. 2012). In addition to increasing the content of both H_2_O_2_ and O_2_^•−^ and also the CAT and SOD activity to keep these free radical scavengers on a non-toxic level (Cembrowska-Lech et al. 2015), KAR_1_ and GA_3_ also increased the content of the non-enzymatic antioxidant, AsA (Fig. 6a) known to react with O_2_^•−^ and H_2_O_2_ (Li et al. 2010). The effect of KAR_1_ and GA_3_ on the AsA content could be associated with the appearance of an additional isoform, APX-3 (Table 4). The increased content of AsA and the increased activity of APX as well as the appearance of its new isoforms were found during imbibition of non-dormant *Pisum sativum* seeds (Wojtyla et al. 2006). The content of endogenous AsA in embryos of KAR_1_- and GA_3_-treated caryopses (Fig. 6a) is probably suitable for germination, since application of AsA (Table 3) delayed germination. The marked reduction of the stimulatory effect of KAR_1_ and GA_3_ (Fig. 5) by lycorine, an inhibitor of AsA biosynthesis may suggest their important mediating role in the response of caryopses to these regulators. AsA can be used not only as a detoxicant, but also as a substrate for dioxygenases which are included in the biosynthesis of gibberellins and ABA (Arrigoni and De Tullio 2002). Suppression of the AsA content by lycorine has been demonstrated to be correlated with reduced germination of rice seed and reduced expression of GAs biosynthesis genes (Ye et al. 2012). Gibberellin biosynthesis has been previously postulated to be required for stimulation of germination in dormant *A. fatua* caryopses by KAR_1_ (Kępczyński et al. 2013). In addition, a high level of ascorbate favoring a lower abundance of ABA and signaling in the control of plant growth and development has been discussed (Foyer and Noctor 2011). AsA is also required for biosynthesis of ethylene (Arrigoni and De Tullio 2002), a hormone required for stimulatory effect of KAR_1_ on germination of dormant *A. fatua* caryopses (Kępczyński and Van Staden 2012). The highest level of AsA in embryos at the end of phase II of KAR_1_- or GA_3_-treated caryopses is probably associated with this compound being required for preparation to cell division and cell expansion. It has been reported before that both the cell division and cell expansion in onion roots require AsA (Arrigoni and De Tullio 2002). Lycorine, an inhibitor of AsA synthesis, was found to block both the cell division and expansion in pea root (Citterio et al. 1994). The stimulatory effect of AsA on cell growth can be associated with its role as a cofactor for enzymes involved in cell wall synthesis (Cooper et al. 1994). KAR_1_ and GA_3_ increased also DHA, but much less effectively than AsA (Fig. 6b). The DHA content has probably to be maintained on a suitable low level, since the compound at low concentrations is known to inhibit activities of several enzymes (Morell et al. 1997) and is responsible for the decrease in mitotic activity (Potters et al. 2000). The progressive increase of AsA during imbibition of caryopses in the presence of KAR_1_ or GA_3_ contributed to the increase in the AsA/DHA ratio (Fig. 6c). The enhancement of the ratio may indicate that KAR_1_- and GA_3_-treated caryopses are prepared to scavenge ROS and also to induce cell division. The AsA/DHA ratio was considered as responsible for the cell cycle in plants (Foyer and Noctor 2011).

### GSSG and GSH contents

Embryos from dry dormant *A. fatua* (Fig. 7a, b ) and barley (Bahin et al. 2011) caryopses showed levels of GSH to be a little or much higher than those of GSSG, respectively. On the basis of increasing glutathione content during after-ripening of barley caryopses, glutathione was suggested to be implicated in the dormancy alleviation (Fontaine et al. 1995). However, the analysis of GSH and GSSG during dry storage of barley grains did not confirm the role of glutathione in dormancy release (Bahin et al. 2011). The data presented show that KAR_1_ and also GA_3_, while stimulating germination of dormant caryopses, decreased the GSSG content markedly in phase II and much less effectively increased that of GSH (Fig. 7a, b ). The increase of GR activity by KAR_1_ and GA_3_ (Fig. 8) seems to be responsible for changes in the content of GSSG (Fig. 7a). The higher level of GSSG in embryos from dry or imbibed dormant caryopses than in embryos from KAR_1_- or GA_3_-treated caryopses may indicate that GSSG at a high concentration is responsible for the lack of germination. GSSG has been suggested to block protein synthesis in dormant wheat embryos (Fahey et al. 1980). Most likely, the endogenous level of GSH is suitable for germination, as the simultaneous application of GSH and KAR_1_ or GA_3_ delayed germination but did not affect the final percentage of germinated caryopses (Table 3).

The increase in the GSH content induced by KAR_1_ and GA_3_ (Fig. 7b) can be associated with an increasing number of cells in phase S (Table 1). The GSH level was found to be low in phase G_1_, while an increase in total GSH is necessary for the transition from phase G_1_ to S in the root meristem (Kerk and Feldman 1995). GSH was found to be recruited into the nucleus at phase G_1_ from cytosol. Comparison of the GR activity and GSH level in KAR_1_- and GA_3_-treated embryos may suggest that the GSH content did not correspond with the GR activity because of its possible participation in regeneration of GSSG in reaction with DHA. Probably therefore embryos from caryopses treated by both KAR_1_ and GA_3_ had a higher content of AsA than DHA. The KAR_1_- and GA_3_-induced increase in the GSH/GSSG ratio may indicate that embryos of *A. fatua* are resistant to potential ROS toxicity. KAR_1_ and GA_3_ increased the GSH/GSSG ratio more effectively than the AsA/DHA (Figs. 6c, 7c), suggesting that the GSH/GSSG ratio is more important for stimulation of germination in dormant caryopses. The increase of both ratios by KAR_1_ and GA_3_ indicates that these caryopses, in contrast to the untreated ones, are prepared to scavenge the ROS excess.

In summary, KAR_1_, and probably GA_3_ also, stimulates the dormancy release and germination of *A. fatua* caryopses by inducing ABA degradation to PA. Both KAR_1_ and GA_3_ are involved in the induction of dormancy release and germination of *A. fatua* caryopses through increase of AsA/DHA and GSH/GSSG ratios as well as DNA replication and β-tubulin accumulation.

#### *Author contribution statement*

JK initiated and designed the research, interpreted the results and wrote the manuscript. DC-L conducted experiments and statistical analysis. All authors read, reviewed and approved the manuscript.

## Abbreviations

ABA: Abscisic acid
APX: Ascorbate peroxidase
AsA: Ascorbate
DHA: Dehydroascorbate
DPA: Dihydrophaseic acid
GR: Glutathione reductase
GSH: Glutathione reduced form
GSSG: Glutathione oxidized form
KAR_1_: Karrikinolide
7′ OHABA: 7′hydroxyABA
PA: Phaseic acid
PC: Plumule+coleoptile
RC: Radicle+coleorhiza
ROS: Reactive oxygen species
